# Burden of Parkinson’s disease in Central Asia from 1990 to 2021: findings from the Global Burden of Disease study

**DOI:** 10.1186/s12883-024-03949-w

**Published:** 2024-11-13

**Authors:** Ruslan Akhmedullin, Adil Supiyev, Rauan Kaiyrzhanov, Alpamys Issanov, Abduzhappar Gaipov, Antonio Sarria-Santamera, Raushan Tautanova, Byron Crape

**Affiliations:** 1 Department of Medicine, Nazarbayev University School of Medicine, Kerey and Zhanibek, Street 5/1, Astana, 010000 Republic of Kazakhstan; 2 Heritage College of Osteopathic Medicine, Ohio University, Athens, USA; 3 Department of Neuromuscular Disorders, University College London, London, UK; 4 School of Population and Public Health, University of British Columbia, Vancouver, BC Canada; 5 Department of Neurosurgery, RSE Medical Centre Hospital of the President’s Affairs Administration of the Republic of Kazakhstan, Astana, Kazakhstan

**Keywords:** Parkinson’s disease, Years lived with disability, Disability-adjusted life years, DALY, Central Asia, Global Burden of Disease

## Abstract

**Background:**

Central Asia is known to face various ecological challenges that constitutes major risk factors for Parkinson’s disease (PD). This study examines the burden of PD in Central Asia, a region where data on neurological disorders is notably sparse.

**Methods:**

Building on the latest Global Burden of Disease Study (GBD 2021), this study investigates the Years of Life Lost (YLLs), Years Lived with Disability (YLDs), and Disability-Adjusted Life Years (DALYs) associated with PD in Central Asia and its countries from 1990 to 2021. The authors calculated average annual percent change (AAPC) to analyze trends, and compared individual country estimates to global figures. Additionally, incorporating data from the World Bank, both Bayesian hierarchical and non-hierarchical frequentist regression models were employed to assess their impact on DALYs.

**Results:**

The DALYs varied across the study period, primarily driven by YLLs. While YLLs showed a uniform trend, YLDs were mostly incremental. Kazakhstan had the highest estimates across all metrics and was the only country aligned with global patterns. Age- and sex-specific estimates revealed substantial variations, with notably high figures found in male subjects from Tajikistan. The YLLs, YLDs, and DALYs for Kazakhstan, Uzbekistan, and Turkmenistan saw a significant increase in AAPCs. In contrast, Kyrgyzstan and Tajikistan saw declines, likely attributable to civic conflict and inter-country differences in population structure. Further comparison of DALY trends revealed significant deviations for all countries from the global pattern.

**Conclusion:**

This study showed an overall increase in PD burden from 1990 to 2021. These findings underscore the need for targeted strategies to reduce PD burden, with a particular focus on Kazakhstan. Integrating historical information is crucial for discussing the plausible mechanisms in studies sourced from the GBD.

**Supplementary Information:**

The online version contains supplementary material available at 10.1186/s12883-024-03949-w.

## Introduction

Neurological disorders are now the leading cause of disability worldwide, and Parkinson’s disease (PD) is the fastest-growing neurodegenerative condition among them [1, 2]. PD is a chronic and non-fatal condition characterized by loss of motor coordination and mobility, especially tremors. Global PD prevalence estimates have doubled over the past 25 years [3], primarily driven by an aging population [1]. The number of patients with PD is expected to rise as population longevity increases, causing an increase in the prevalence of advanced PD [4, 5]. Hence, the projected increase in population life expectancy in the following decades [6] will presumably bring about additional health, social and economic challenges due to PD globally [7], highlighting the need for further improvements in preventive and treatment strategies.

Based on the United Nations categorization of countries, Central Asia comprises of Kazakhstan, Kyrgyzstan, Turkmenistan, Uzbekistan and Tajikistan, all previously part of the Soviet Union [8]. At present, data on PD are especially scarce in low and middle-income countries, which limits the understanding of contributing factors in PD in those regions [9, 10]. While abundant data are available from high-income countries, historical data from developing regions like the five Central Asian countries remains scarce [11, 12]. This region shared a common culture, lifestyle, and healthcare system for seven decades. Since the dissolution of the Soviet Union in 1991, the five countries have undergone major reforms in healthcare, social, and economic systems [13, 14], which has the potential to affect the burden of chronic diseases.

There has been a scarcity of analysis of the burden of PD in these Central Asian countries, over the past 30 years of independence. The existing evidence suggests that pesticides, low-frequency magnetic fields, solvents, heavy metals, chemicals, and air pollutants are significant risk factors for PD [15, 16]. Historically, Central Asian region, Kazakhstan in particular, served as a nuclear testing site for the Soviet Union, and has experienced significant ecological challenges related to the Aral Sea shrinkage [17], Baikonur cosmodrome spacecraft launch site activities [18], and natural disasters, which may be related to the risk factors for PD [5]. Furthermore, the region is one of the major cotton producers worldwide, subjecting people to chronic pesticide exposure [19]. The cotton industry in Central Asia employs millions of people, making it the largest seasonal activity in the world [20, 21]. The uncommon exposures related to these environmental factors, and the sparsity of research on PD in Central Asia calls for further epidemiological studies on PD in this region.

This study evaluates the burden of PD in Central Asia, a region where data on PD burden are notably sparse. Utilizing publicly available data from the Global Burden of Diseases 2021 (GBD 2021), our study provides estimates of disability-adjusted life-years (DALYs) by sex and nation from 1990 to 2021 in Central Asia, and regression analyses conducted to assess the impact of historical factors on these DALYs.

## Methods

### Overview and definitions

This study analyzed the burden of PD in Central Asia based on the GBD 2021, covering all these five countries. In the GBD 2021, 204 nations and territories’ population sizes, morbidity rates, and mortality rates were estimated using a consistent and comparable methodology. The general information is available elsewhere [9, 22].

The International Classification of Diseases tenth revision (ICD-10) codes for PD were G20, G21, and G22. The International Parkinson and Movement Disorder Society defines the core feature of PD as motor Parkinsonism, which is characterized by bradykinesia along with rest tremor or rigidity [23].

## Data sources and measures

We utilized publicly available data from the GBD 2021, where vital registration systems, including records of births and deaths, served as the main data source for the PD burden [24, 25]. Additional demographic data on share of the population aged 65 and above, population size, crude mortality rate, life expectancy, completeness of death registration, and employment in agriculture were derived from the World Bank databases [26]. Using data from the GBD for 1990 to 2021, the average annual percent change (AAPC) in trends was calculated for years of life lost (YLLs), years lived with disability (YLDs), and disability-adjusted life years (DALYs). AAPC represents a summary measure of the trend over a specified time-period, which is calculated as a weighted average of the APCs from the joinpoint model, with the weights equal to the length of the APC interval. All estimates were reported per 100,000 population. The 2.5th and 97.5th percentiles of an ordered set of 1000 draws were used to calculate the 95% uncertainty interval (UI). PD severity was defined using the HOEHN and YAHR stages [27]. YLDs were prevalence-based and calculated by multiplying the corresponding disability weights. YLLs were calculated by multiplying the number of deaths in each age group by the remaining life expectancy in that age group. DALYs are the sum of YLLs and YLDs. Age-standardized populations were calculated using the GBD standard life table. We further enhance our analysis by using a Bayesian framework to estimate the relationship between historical demographic data and DALYs.

### Statistical analysis

We conducted a descriptive data analysis, including age-adjusted DALY rates for PD across Central Asia and compared these estimates with global trends. Piecewise regression models were utilized to fit the regression models and identify any significant changes in trends. Given the long period analyzed, we fitted a model with zero breakpoints to reflect the overall trend from 1990 to 2021, as the data suggested a relatively constant trend throughout this period. We evaluated the fit of these models using Bayesian Information Criterion (BIC) and confirmed that a model with zero joinpoints was appropriate based on the observed data patterns. The AAPC models were fitted as log-linear models, assuming a constant rate of change over time with uncorrelated errors. Acknowledging between-country variations in the region, Bayesian hierarchical regression models were chosen for their ability to account for both individual country-level effects and overall regional trends, which were fitted to demographic parameters using the probabilistic language Stan to estimate the association between predictors and DALYs. In Bayesian statistics, we utilize prior distributions, likelihood functions, and posterior distributions to incorporate existing knowledge and update our beliefs based on observed data. We used uniform priors for all the model parameters. For the model, four chains of 4000 Markov Chain Monte Carlo samples were retrieved. The first 800 samples were discarded as the warm-up. Model convergence was assessed using the R-hat diagnostic statistics, with values close to 1 indicating convergence. We also examined the effective sample size statistic, and analyzed trace plots to ensure that the chains adequately explored the parameters. General information on the methods utilized is available elsewhere [28]. Separate single-level frequentist regression models were provided for each location and for each demographic factor. P-values are two-sided and considered statistically significant at < 0.05 for demographic factors (proportion of the population aged 65 and above, year, population size, life expectancy, and employment in agriculture) as well as for AAPCs in our analyses. All analyses were performed using R software (version 4.3.1).

## Results

**Fig. 1:**
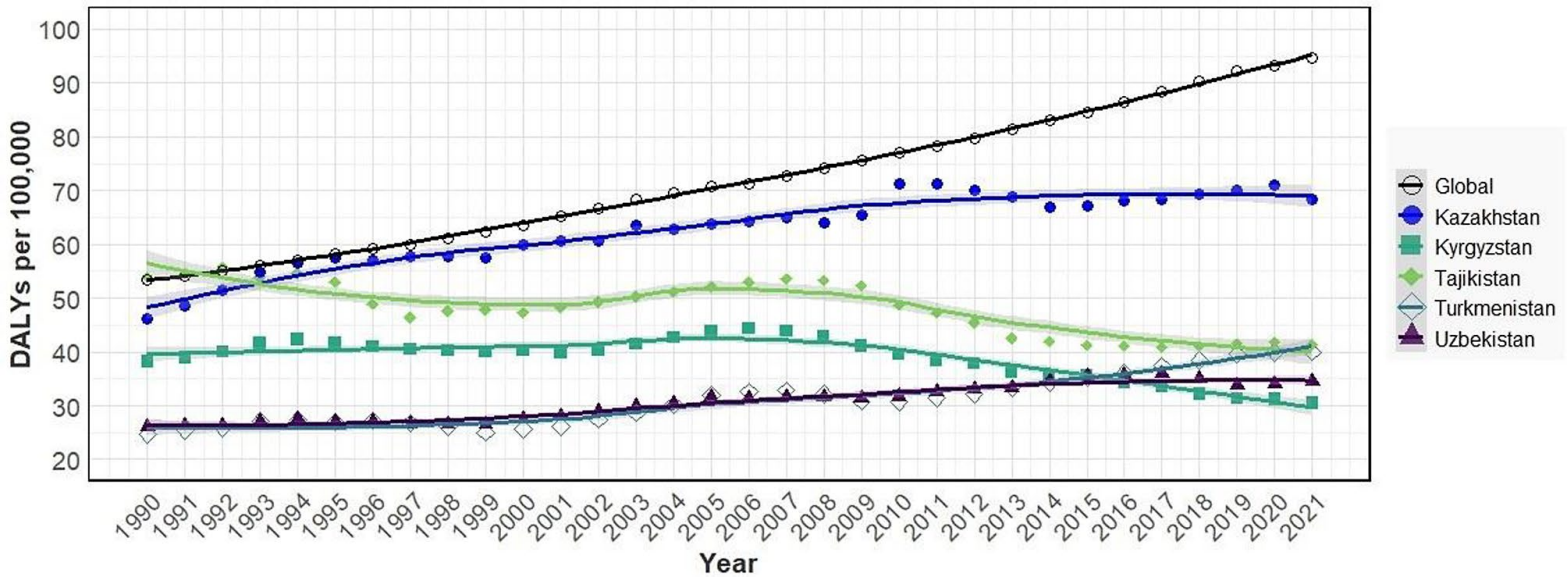
Trends in disability-adjusted life years of Parkinson’s disease per 100,000 population for both sexes in Central Asian countries and World during 1990–2021

The burden of PD, in terms of DALYs, showed significant variation across Central Asia during the study period. Although YLDs were somewhat stable during observation, YLLs revealed minor variations for all specified locations, contributing substantially to DALYs. Generally, the highest contribution of both YLLs and YLDs to DALYs was observed at the age of 65 and above. For all locations, PD was more burdensome in older age groups and more distinct in male subjects (Supplemental Figs. 1–3). Kazakhstan consistently recorded higher crude estimates for all metrics in the region (Fig. 1, Supplemental Fig. 4). In age-and sex-specific estimates, however, the analysis showed substantial variations in DALYs, with the highest estimates being observed in male subjects from Tajikistan, particularly during 1990–1997, followed by Kazakhstan (Supplemental Figs. 1–3). The estimates for three other Central Asian countries were similar to each other. While YLLs displayed a somewhat flat trend, YLDs showed an upward trend in most countries (Supplemental Figs. 1 and 2).

For the AAPCs, PD-related YLLs, YLDs, and DALYs trends varied over the study period (Table 1). Globally, there was a significant increase in YLLs, YLDs, and DALYs, with AAPCs of 1.64%, 3.00%, and 1.90%, respectively (Table 1). Kazakhstan was the only country following a comparable pattern, experiencing increases in all metrics, with an AAPC of 1.06% for YLLs, 1.33% for YLDs, and 1.17% for DALYs, while Uzbekistan and Turkmenistan showed more modest growth. In contrast, Kyrgyzstan and Tajikistan saw declines in the YLLs and DALYs. Kyrgyzstan’s YLLs fell by 0.89% per year and DALYs fell by 0.78%, while Tajikistan demonstrated a 1.08% annual decline in YLLs and a 0.89% decrease in DALYs. However, the YLDs in Tajikistan showed a modest increase (0.44%). Additionally, further comparison of DALYs trends between the global data and each country individually also revealed significant differences, with consistent deviations from global trends (Supplemental Fig. 5).

**Fig. 2:**
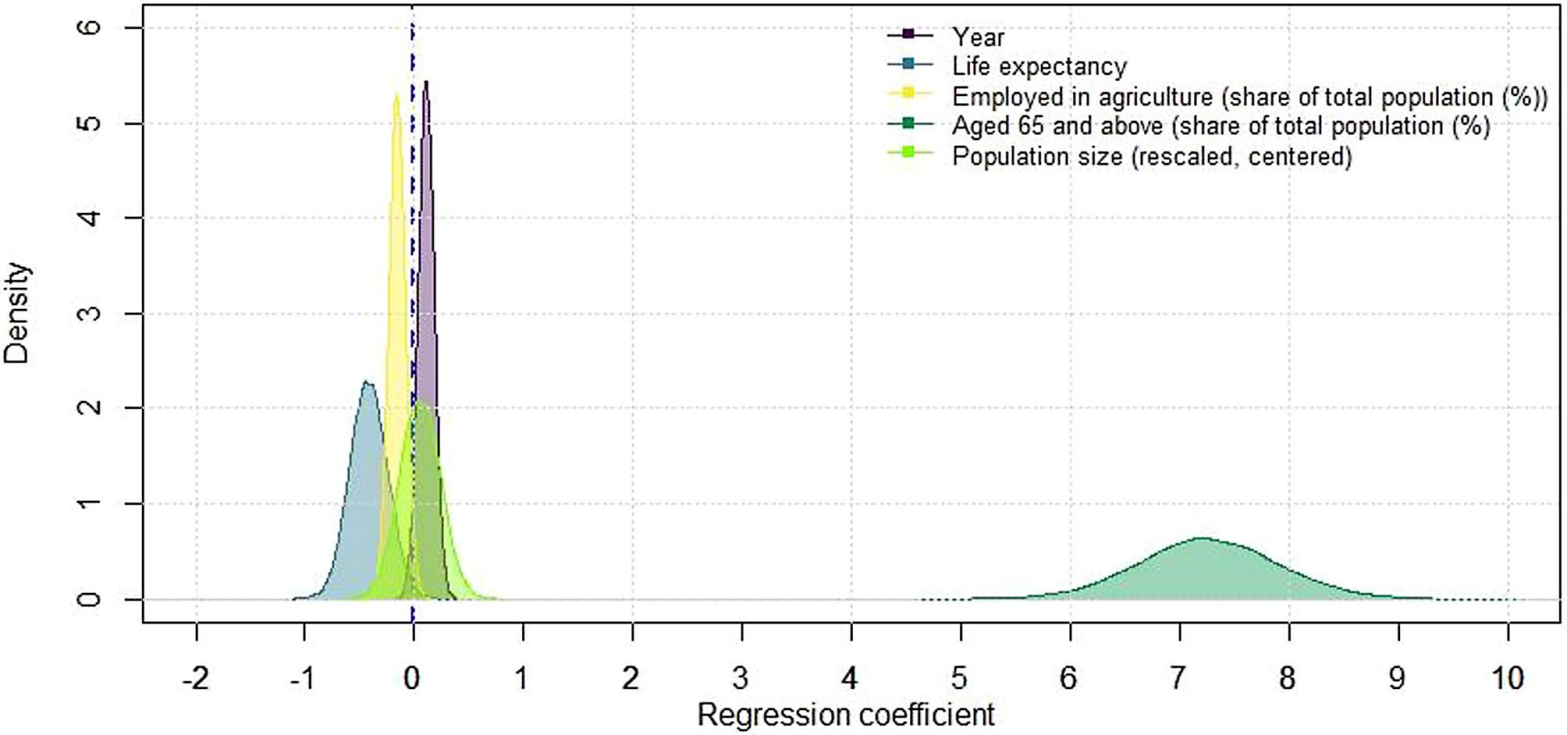
Bayesian posterior distributions of regression coefficients. *The population size has been rescaled to millions and centered around the mean

The estimates for incidence, prevalence, and mortality rate were aligned with DALYs and showed an increase in Central Asian countries (Supplemental Tables 1 and Supplemental Figs. 6–8). Further hierarchical Bayesian regression analysis revealed increase in coefficients for the year (0.11; 95% CrI: -0.03; 0.25), for the share of population aged 65 and above (7.26; 95% CrI: 6.01; 8.53), and population size (0.05; 95% CrI: -0.32; 0.41). In contrast, the coefficients decreased for life expectancy (-0.42; 95% CrI: -0.76; -0.07) and employment in agriculture (-0.16; 95% CrI: -0.30; -0.01). Alternatively, the posterior probabilities of exceeding the null value for the modeled coefficients were 94%, 100%, 61%, 0.9%, and 2% for the year, population aged 65 and above, population size, life expectancy, and employment in agriculture, respectively (Fig. 2). Although separate frequentist single-level regression models revealed variations between countries, most coefficients aligned with those from the Bayesian model (Supplemental Fig. 9).

**Table 1:** YLLs, YLDs, and DALYs for Parkinson’s disease for both sexes in 1990 and 2021, and average annual percent change during 1990–2021 in Central Asia

	YLLs (95% UI)				YLDs (95% UI)			DALYs (95% UI)		
	1990	2021		AAPC 1990–2021 (95% CI)	1990	2021	AAPC 1990–2021 (95% CI)	1990	2021	AAPC 1990–2021 (95% CI)
Global	45.06 (41.69; 48.06)	73.51 (66.57; 79.07)	1.64 (1.62; 1.67)**		8.44 (5.94; 11.26)	21.17 (14.87; 27.97)	3.00 (2.96; 3.04) **	53.51 (49.54; 57.55)	94.68 (85.37; 103.11)	1.90 (1.88; 1.92) **
Kazakhstan	38.53 (36.28; 41.06)	57.24 (50.91; 63.97)	1.06 (0.84; 1.28)**		7.67 (5.34; 10.59)	11.20 (7.62; 15.08)	1.33 (1.22; 1.44) **	46.20 (42.88; 50.12)	68.44 (61.48; 75.50)	1.17 (0.93; 1.28) **
Uzbekistan	20.09 (16.13; 25.99)	26.26 (22.92; 30.01)	1.12 (0.95; 1.29)**		5.92 (4.09; 8.23)	8.22 (5.86; 11.36)	1.12 (0.93; 1.30) **	26.02 (21.05; 32.72)	34.48 (30.21; 39.05)	1.12 (1.00; 1.25) **
Kyrgyzstan	31.85 (27.84; 37.02)	24.39 (20.83; 28.29)	-0.89 (-1.26; -0.52)		6.32 (4.40; 8.68)	6.06 (4.18; 8.17)	-0.26 (-0.39; -0.13) **	38.18 (33.49; 43.35)	30.45 (26.49; 34.93)	-0.78 (-1.08; -0.49) **
Tajikistan	48.35 (35.23; 74.30)	35.05 (28.91; 41.90)	-1.08 (-1.36; -0.80) **		5.50 (3.88; 7.81)	6.31 (4.32; 8.68)	0.44 (0.31; 0.56) **	53.85 (40.08; 79.78)	41.36 (35.16; 48.58)	-0.89 (-1.14; -0.64) **
Turkmenistan	20.40 (19.28; 21.44)	33.41 (26.31; 41.12)	1.52 (1.30; 1.74) **		4.26 (2.93; 5.96)	6.54 (4.55; 8.77)	1.58 (1.40; 1.76) **	24.67 (22.91; 26.75)	39.96 (32.95; 48.04)	1.53 (1.33; 1.73) **

YLDs- Years lived with disability; YLLs- Years of life-lost; DALYs- Disability-adjusted life years; AAPC- Average annual percent change; ** p-value < 0.001

## Discussion

The findings of our study revealed that PD burden increased in AAPCs over 1990–2021 for most Central Asian countries, with Kazakhstan having the highest figures in Central Asia. These results were consistent with previous studies [9, 25]. Unlike previous GBD studies, the present study aimed to provide a unique geographical focus and went through the historical and regional features in the evaluation of trends for PD burden. In addition, we identified significant associations between historical factors and DALYs.

Given that the incidence, prevalence, and mortality estimates are integral to the DALYs, their trends were closely aligned across the study period. A systematic review highlighted a female predominance for prevalent PD in Central Asia [11]; however, the contribution of males to DALYs has been substantially greater. In Central Asia, Kazakhstan saw the most pronounced burden of PD throughout the study period, which complies with the WHOs mortality estimates [29], where neuropsychological conditions were reported to be the highest death contributor. On the other hand, Kazakhstan launched the development of a large healthcare database to track the epidemiology of chronic diseases in 2005 [30], and was the pioneering country in Central Asia that became a member of the International Parkinson Disease Genomics Consortium [31]. Such initiatives could improve PD identification, which is a plausible explanation for the country’s elevated burden.

The burden of PD is less pronounced in individuals younger than 65 years. Our differences both within and between locations may partly reflect the consistently lower share of the population aged 65 and above in all Central Asian countries, except Kazakhstan, despite comparable life expectancy (Supplemental Fig. 10). Thus, a reduction in the proportion of elderly people could naturally lead to a lowered overall burden of PD as the disease normally affects older individuals. However, between 1990 and 2021, Kazakhstan experienced the lowest population growth of 15%, compared to 40% in other Central Asian countries (Supplemental Fig. 11). Such a demographic transition toward a younger population may affect the epidemiology of PD. Variations could also indicate a combination of factors, such as mortality patterns or historical events, affecting population structure. Likewise, the exceptional estimates for Tajikistan most likely accounted for the Civil War from 1992 to 1997 [32], which began right after the country gained independence from the Soviet Union. This resulted in the collapse of the healthcare system, destruction of living conditions, and demographic consequences, which steeply increased mortality estimates (Supplemental Figs. 12 and 13). Nevertheless, the reasons for discrepancies are still likely to be multifactorial, including variations in access to healthcare or differences in healthcare-seeking behavior, underdiagnosis/misdiagnosis due to differences in symptom presentation, and healthcare utilization. The accuracy of PD diagnosis remains a challenge, particularly during the early stages of PD, which may contribute to the discrepancies observed across metrics [33]. In addition, the lowered completeness of death registration with cause-of-death information could also contribute to differences within Central Asia (Supplemental Fig. 14). This suggests that a large proportion of patients could still be misdiagnosed/undiagnosed and/or missed for the global burden estimations.

A previous GBD study attributed the increase in PD burden to rising life expectancy [9], however, our findings suggest that factors such as improved diagnosis and healthcare access may play a more significant role. However, the observed discrepancies in regression coefficients could indicate aggregation errors or methodological differences between the GBD and the World Bank [26]. Our analysis based on aggregated data, it may obscure individual-level variations, further suspecting ecological fallacy. Instead, awareness of diagnosis and better access to treatment for PD patients are plausible explanations [33]; however, notable proportion of PD cases in Central Asia remains undetected [31]. The elevated burden in male subjects may reflect higher occupational factors, which has been consistently reported previously [24, 25]. In addition to aging, PD is associated with environmental risk factors such as agricultural pesticide exposure [5, 10]. While previous studies have associated PD with agricultural pesticide exposure, our findings suggest a negative association between agricultural employment and DALYs, possibly reflecting changes in the population structure or reporting practices (Figs. 2 and 9; Supplemental Fig. 15). Although it is encouraging to highlight such an association, it is worth noting that suspicions over temporality and the biological gradient still concern this matter. That is, employment in agriculture itself might not reflect the pesticide exposure level, and the duration of exposure among agricultural workers might be less than that in other cohorts, further indicating population fallacy. In addition, variations in environmental regulations and agricultural practices can account for the direction of this association. Nevertheless, evidence to conclude a causal relationship between particular pesticide compounds or combined pesticides is limited [34]. This highlights the focus of current research on the interactions between genetic susceptibility and environmental exposure [35].

Discrepancies were observed between the hierarchical and non-hierarchical model estimates. A coefficient for population size reported an increase in DALYs; however, population growth would mean a decrease in the share of older groups in the demographic structure, which should reduce the overall burden. In this sense, the single-level model seems natural, showing a decrease. In contrast, the coefficient estimates for the share of the population aged 65 years and older were associated with a decrease in DALYs for certain countries, further favoring the hierarchical model. Regardless of the obvious similarities among Central Asian countries, there are significant internal variations as well as inter-country differences, which have likely increased since the dissolution of the Soviet Union. These factors may explain the lack of homogeneous patterns of PD. Further research is required to explore the temporal relationships between these factors and methodological approaches.

Finally, we observed differences between locations over time for all the metrics. These observations could be driven by changes in various factors and circumstances. While GBD studies utilize hierarchical meta-regression tools, standardizing estimates from all locations, they typically do not provide a thorough and detailed discussion of their findings. This is reasonable given that hundreds of diseases and risk factors are covered at once. On the other hand, a lack of historical factors might limit the exploration of interplaying factors and the provision of plausible mechanisms for the observed trends. DALYs are the most commonly employed primary endpoint; however, diseases at late stages/ages contribute substantially to DALYs, particularly for chronic diseases in developing regions, where life expectancy is lower. Current estimates for the GBD study were developed based on incomplete data and models, which were improved in each GBD study [9]. Hence, the need for separate disease groups and regional analyses is warranted. Our study emphasizes the need to consider demographic and historical determinants to critically test and discuss the plausibility of the findings in future studies departing from GBD iterations.

### Limitations

Our study has several limitations. The study departed data from the GBD iteration that standardized and extrapolated estimates from all locations, which could result in deviations in the burden estimates This study is based on aggregated data from the GBD and the World Bank, assuming associations at the highest level that may not reflect underlying relationships, indicating ecological fallacy. Furthermore, variations in the statistical models yielded different results, affecting the interpretation of how population size or age structure relates to DALYs. In addition, employment in agriculture was used as a proxy variable to evaluate the association between pesticide exposure and the DALYs. Therefore, any observed associations should be interpreted with caution. More research could prove or disprove the direction of the association by examining the ways in which these parameters may affect DALYs. Nevertheless, we attempted to minimize these limitations by narrowing the geographical focus and using analytical approaches that were consistent with prior studies specifically on PD. Historically, labor migration has been a widespread response to the challenges of unemployment and poverty in Central Asia. It is also plausible that some incident and prevalent PD cases were not identified, especially during the early post-Soviet Union period when healthcare facility availability was extremely limited. Although such migration and healthcare shortages could result in the loss of many PD cases, coverage of a large period of time is assumed to catch systematic patterns and lower their impact on the findings. The burden of PD is pronounced in late ages, and one of the main limitations in PD epidemiology is related to challenges in the proper diagnosis of co-occurring chronic conditions that affect survival, causing biases in cause-specific mortality, quality of life, and disability estimates in different regions. Finally, we conducted a descriptive study, and our findings are exploratory, requiring further confirmation, although we tried to encompass a large period and employed recognized analyses. Nevertheless, alternative approaches could still be applied to include reasonable predictors to estimate trends or differences; however, it is unlikely that this would substantially alter the main findings.

## Conclusion

This study provides critical insights into the trends and burden of PD in Central Asia from 1990 to 2021. The trends for DALYs decreased for certain countries, which is controversial given global patterns. Further research is required to investigate this discrepancy. Our findings highlight the necessity for more effective strategies to control the burden of PD, particularly in Kazakhstan, where the population structure is comparatively older. In addition, it is suggested that the integration of historical information is crucial for discussing plausible mechanisms in further studies sourced from GBD iterations.

## Electronic supplementary material

Below is the link to the electronic supplementary material.

Supplementary Material 1

## Data Availability

No datasets were generated or analysed during the current study.
