# Supplementary material for: Burden of Parkinson’s disease in Central Asia from 1990 to 2021: findings from the Global Burden of Disease study
Source: BMC Neurol. 2024 Nov 13;24:444. doi: 10.1186/s12883-024-03949-w (PMC11558836; doi:10.1186/s12883-024-03949-w)
Supplement: Supplementary file 1 — Supplementary Material 1 [file 12883_2024_3949_MOESM1_ESM.docx]

**Burden of Parkinson's disease in Central Asia from 1990 to 2021: Findings from the Global Burden of Disease Study**

**Authors:** Ruslan Akhmedullin^1^, Adil Supiyev^2^, Rauan Kaiyrzhanov^3^, Alpamys Issanov^4^, Abduzhappar Gaipov^1^, Antonio Sarria-Santamera^1^_,_ Raushan Tautanova^5^ and Byron Crape^1^

**Affiliation:**

1. Department of Medicine, Nazarbayev University School of Medicine, Astana, Kazakhstan;
2. Department of Medical Epidemiology and Biostatistics, Ohio University, Athens, USA;
3. Department of Neuromuscular Disorders, University College London, London, UK;
4. School of Population and Public Health, University of British Columbia, Vancouver, BC, Canada;
5. Department of Neurosurgery, RSE Medical Centre Hospital of the President's Affairs Administration of the Republic of Kazakhstan, Astana, Kazakhstan;

*Author to whom correspondence should be addressed.

Corresponding author: Ruslan Akhmedullin, MPH., Research Assistant, Department of Medicine, Nazarbayev University School of Medicine. Address: Kerey and Zhanibek Street 5/1, Postal code 010000, Astana city, Republic of Kazakhstan. Phone: +77026541626. Email: [ruslan.akhmedullin@nu.edu.kz](mailto:ruslan.akhmedullin@nu.edu.kz)

The authors to give readers additional information about their work.

**SUPPORTING INFORMATION.**

**Supplemental Figure 1. Trends Years Lived with Disability from 1990-2021.**


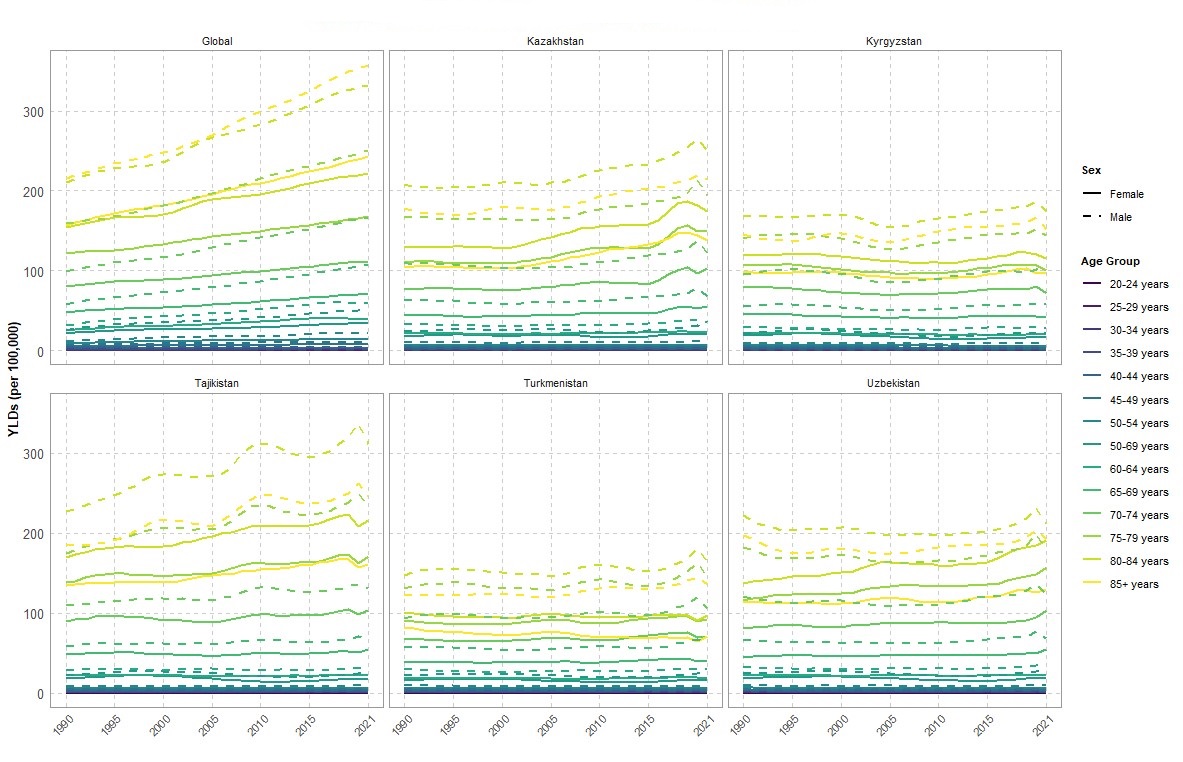


**Supplemental Figure 2. Trends in Years of Life Lost from 1990-2021.**


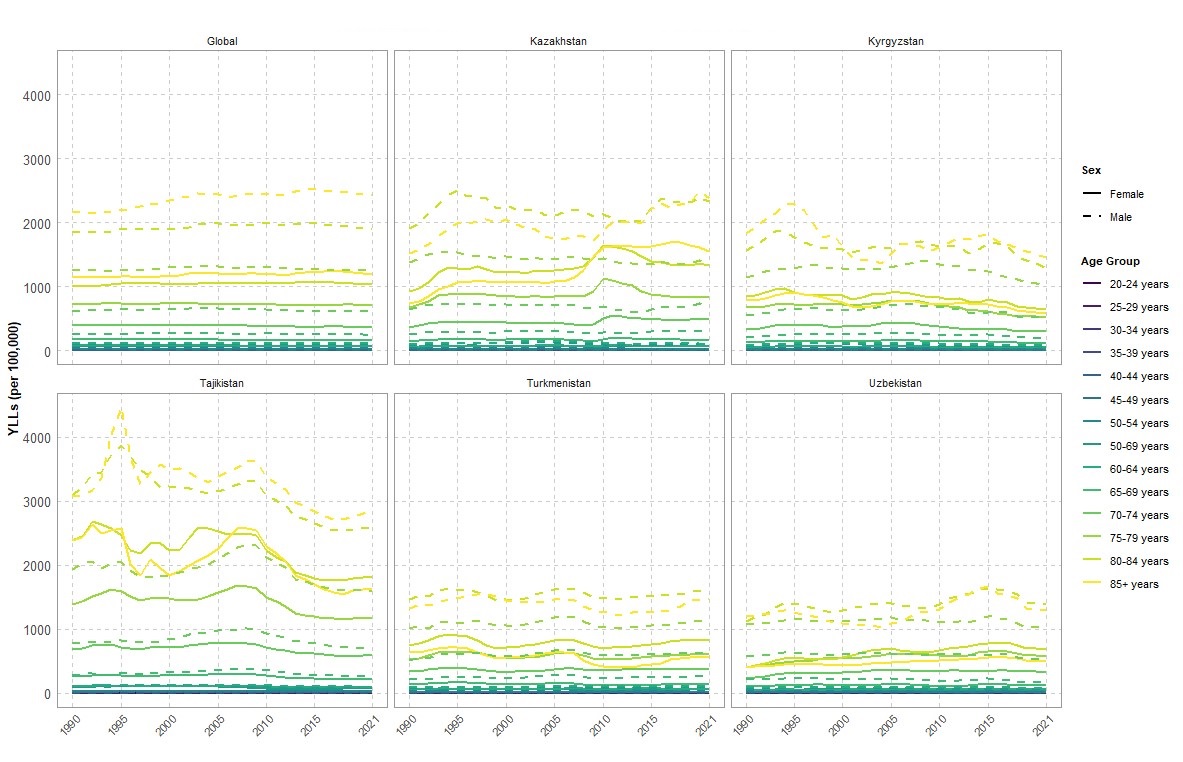


**Supplemental Figure 3. Trends in Disability-adjusted life years from 1990 to 2021.**


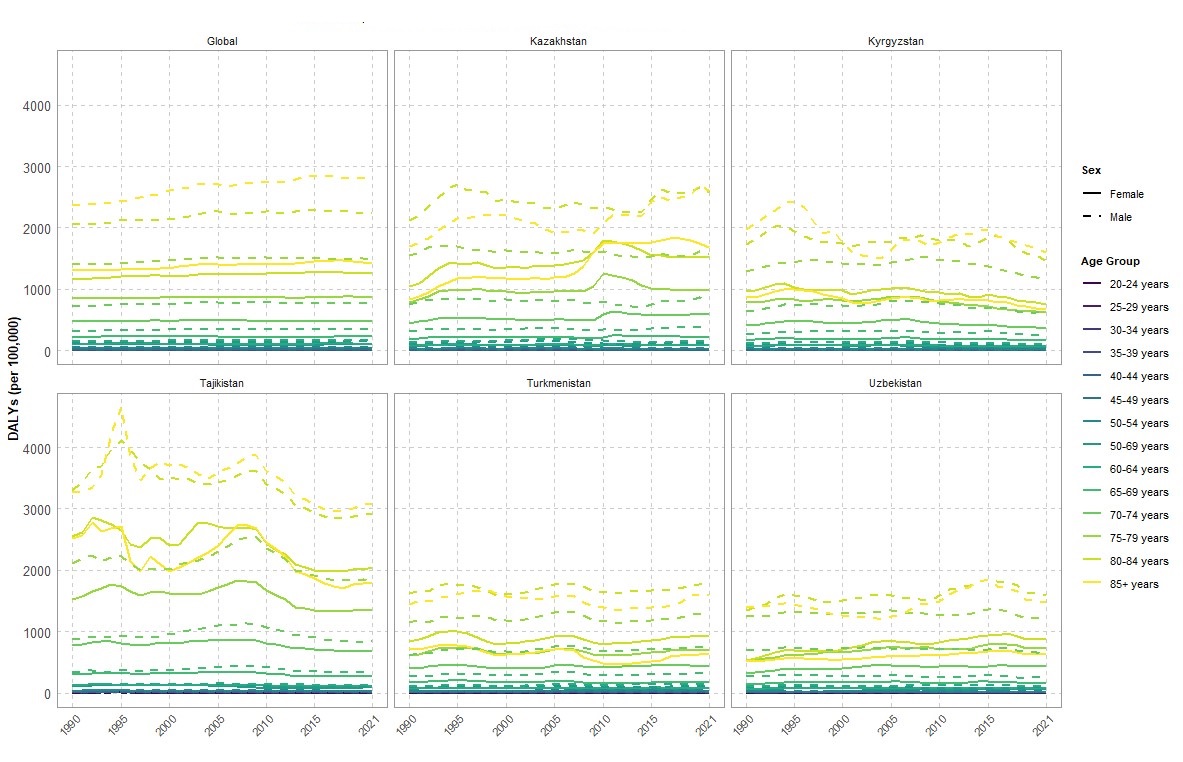


**Supplemental Figure 4. Trends in Years Lived with Disability (A) and Years of Life Lost (B) from 1990 to 2021.**


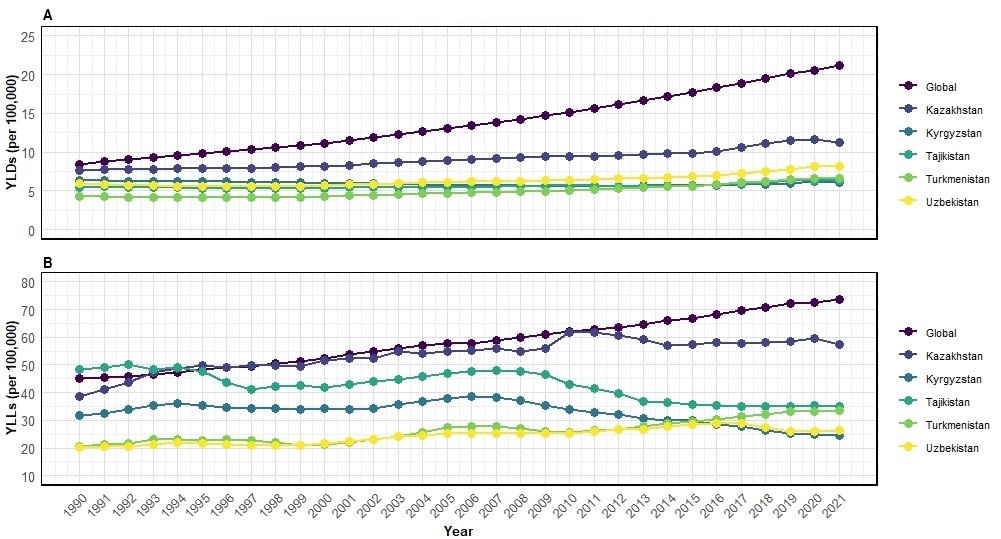


**Supplemental Figure 5. Comparative Analysis of DALYs (Global vs. Individual Countries)**


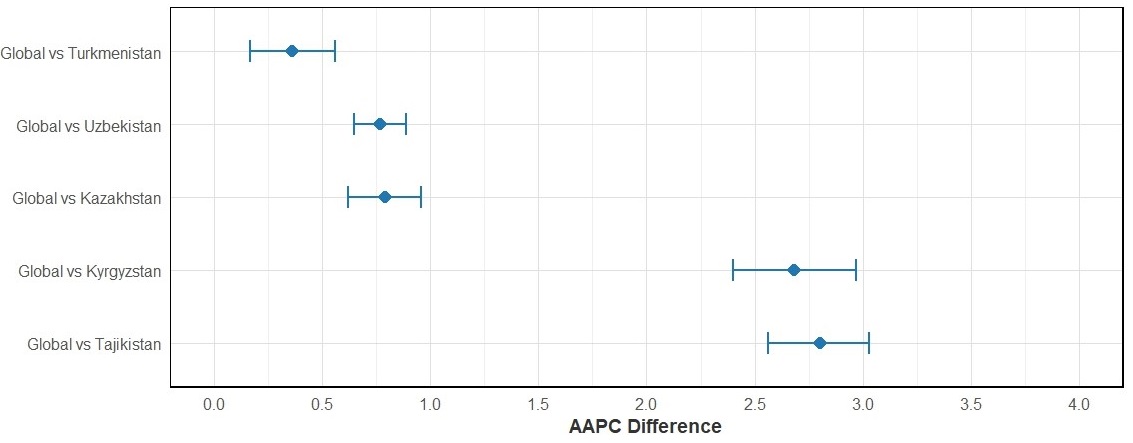


**Supplemental Figure 6. Trends in Incidence Rates from 1990 to 2021.**


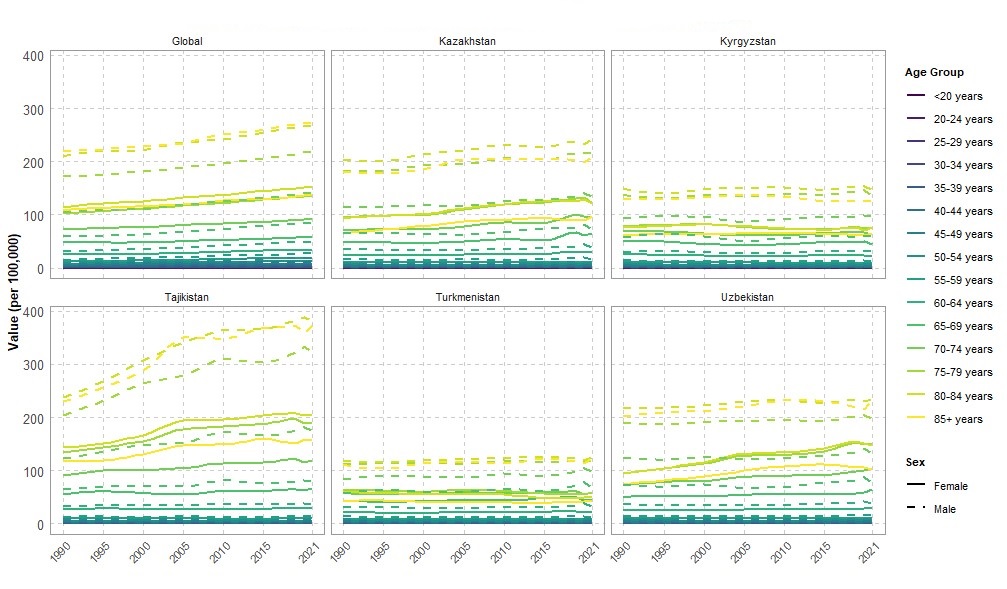


**Supplemental Figure 7. Trends in Prevalence Rates from 1990 to 2021.**


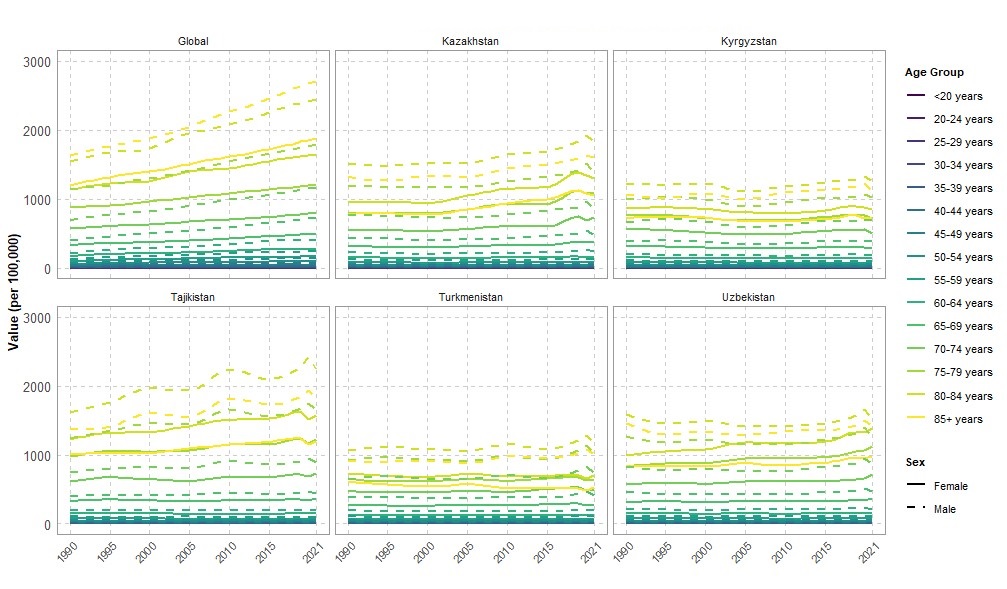


**Supplemental Figure 8. Trends in Mortality Rates from 1990 to 2021.**


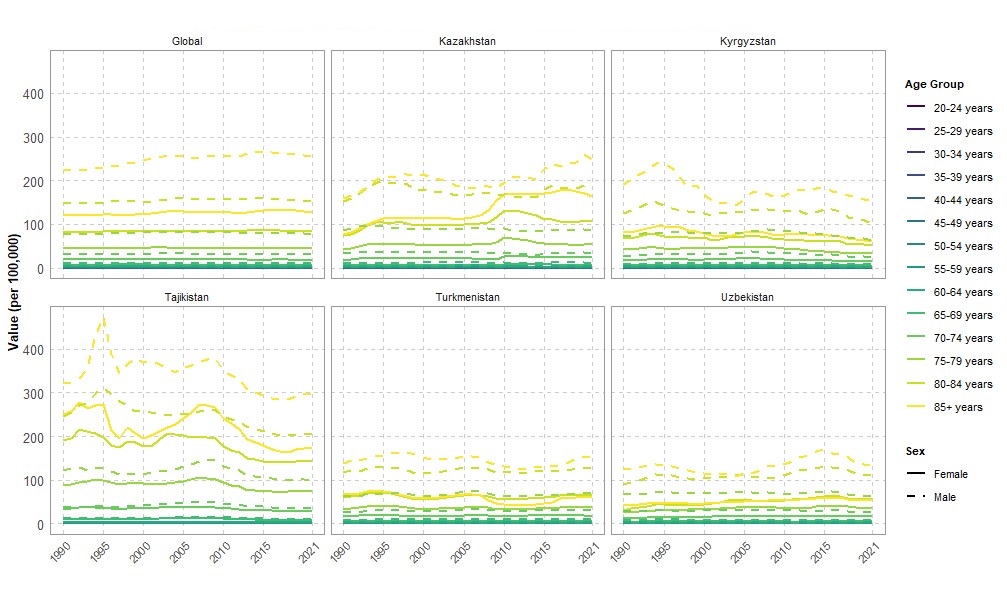


**Supplemental Figure 9. Frequentist Single-level regression models**


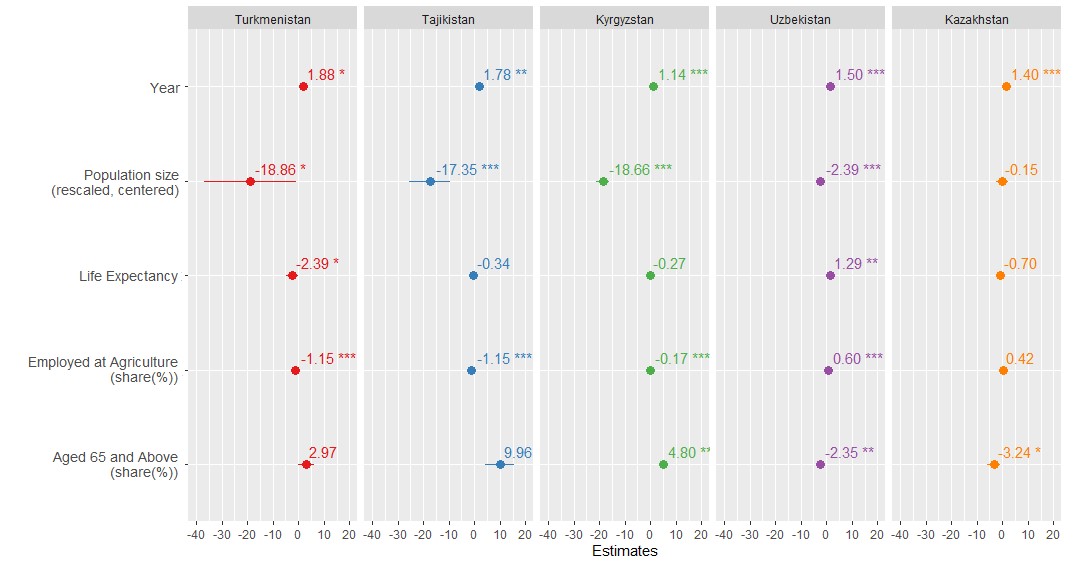
*Significance codes: ***= 0.001; **= 0.01; *= 0.05.*

**Supplemental Figure 10. Share of population aged 65 and above (%) from 1990 to 2021**


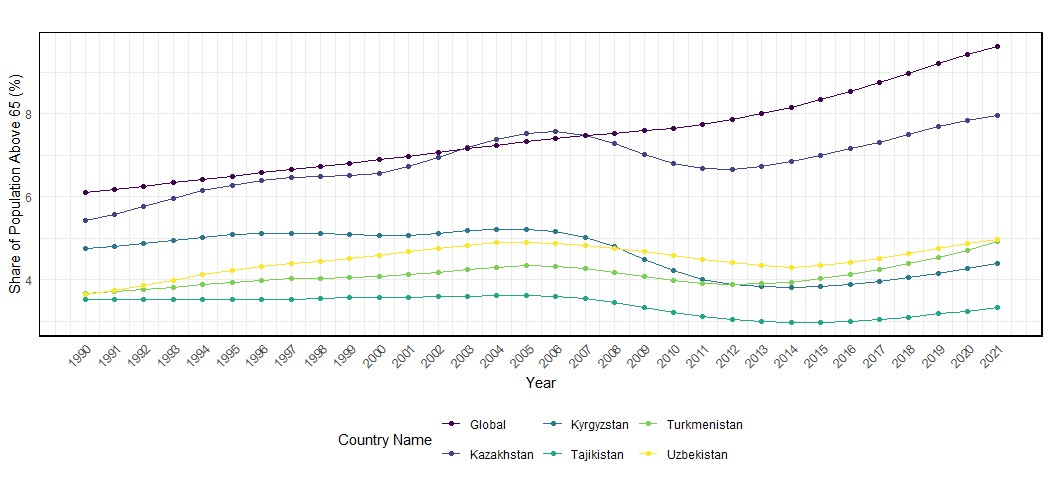


**Supplemental Figure 11. Total Population size from 1990 to 2021**


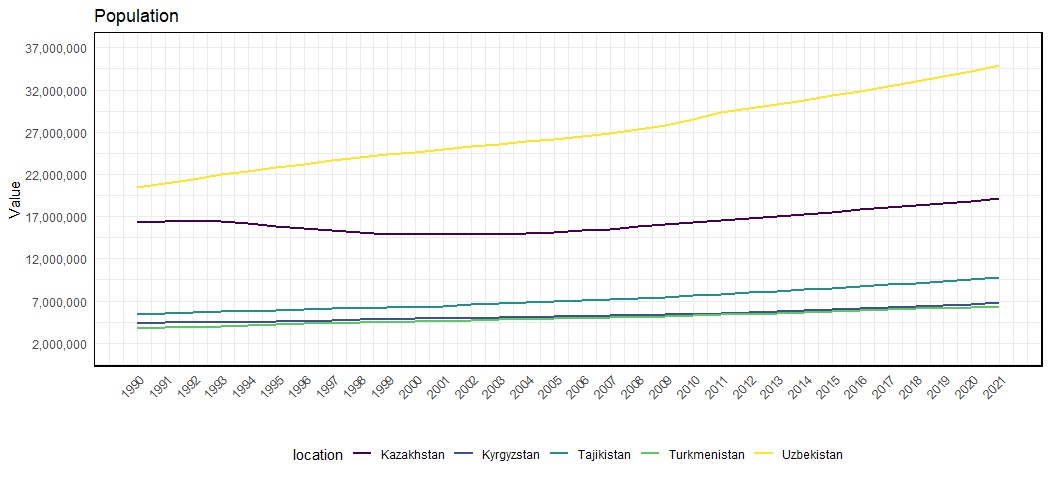


**Supplemental Figure 12. All-Cause crude mortality rate (per 1000) from 1990 to 2021**


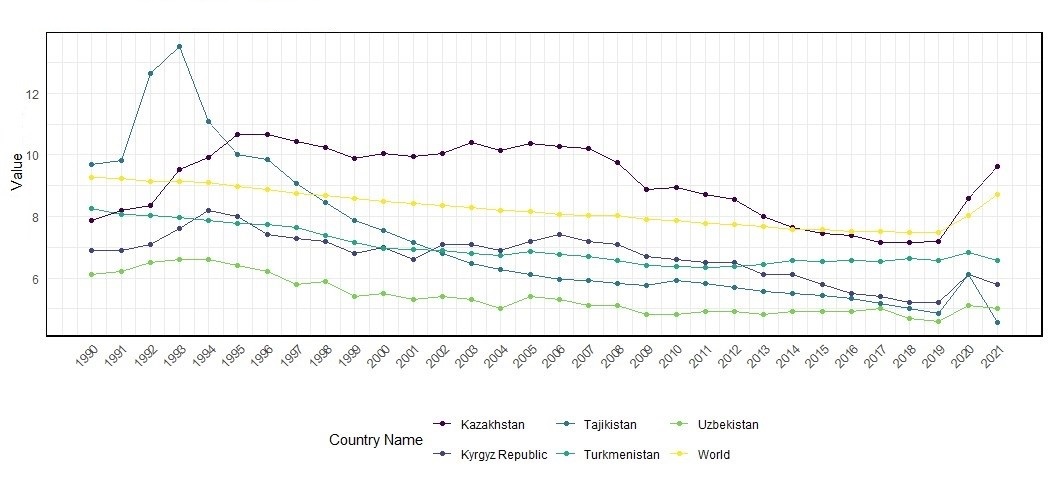


**Supplemental Figure 13. Life Expectancy at birth from 1990 to 2021**


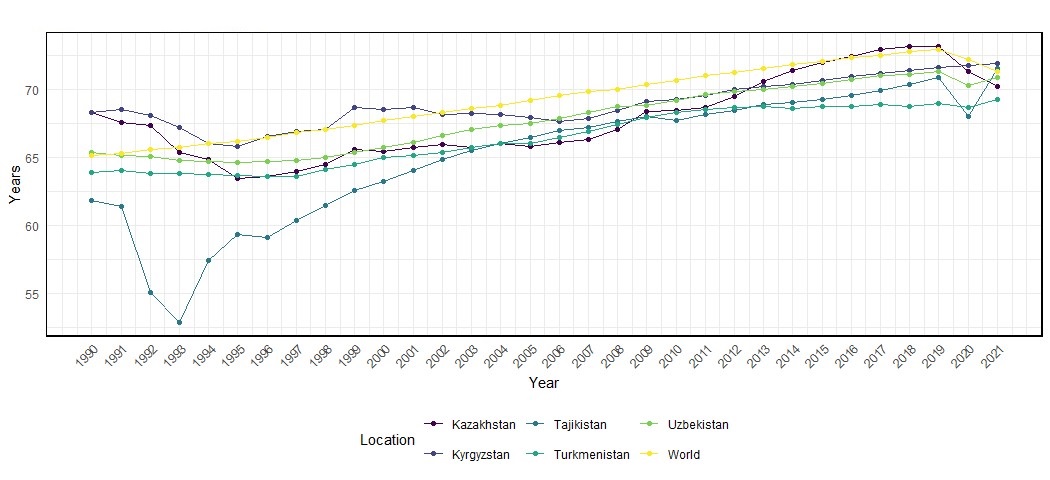


**Supplemental Figure 14. Completeness of death registration with cause-of-death information (percent)**


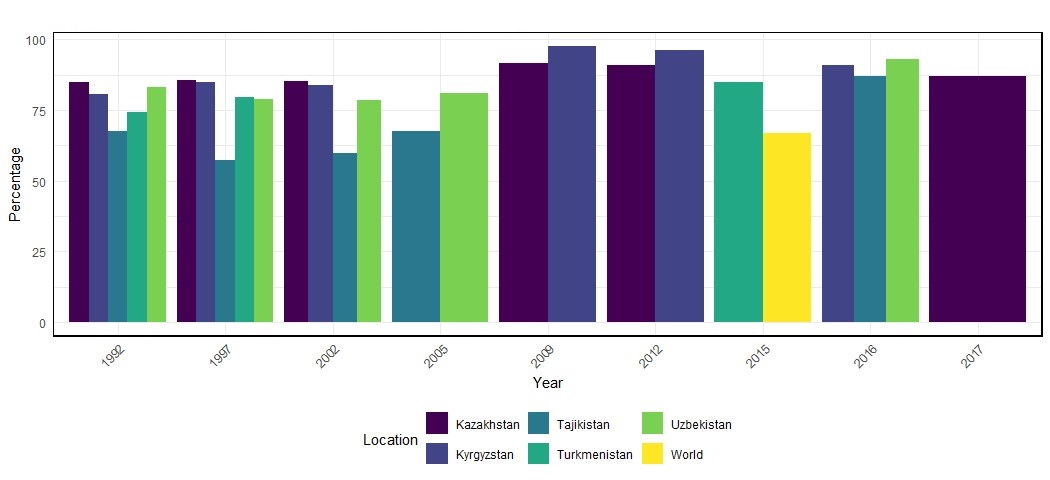


**Supplemental figure 15. Share of total population employed in agriculture from 1990 to 2021**


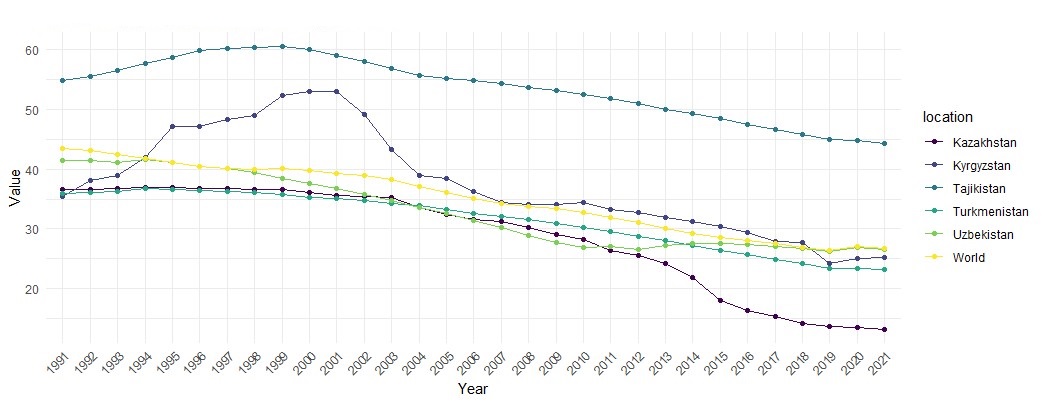


Supplemental Table 1. Epidemiology (per 100,000) for Parkinson’s disease for both sexes from 1990 to 2021 in the World and Central Asia.

|  | **Incidence (95% UI)** | | | **Prevalence (95% UI)** | | | **Mortality (95% UI)** | | |
| --- | --- | --- | --- | --- | --- | --- | --- | --- | --- |
|  | **1990** | **2021** | **AAPC (95% CI)** | **1990** | **2021** | **AAPC (95% CI)** | **1990** | **2021** | **AAPC (95% CI)** |
| Global | 7.82 (6.95; 8.75) | 16.92 (15.16; 18.82) | 2.55 (2.49; 2.61)** | 59.03 (51.55; 68.05) | 149.12 (132.27; 170.06) | 3.02 (2.98; 3.06)** | 2.78 (2.55; 2.97) | 4.92 (4.40; 5.29) | 1.94 (1.90; 1.98)** |
| Kazakhstan | 7.23 (6.29; 8.19) | 10.83 (9.64; 12.03) | 1.39 (1.32; 1.45)** | 53.50 (43.80; 64.12) | 78.33 (65.90; 90.45) | 1.35 (1.24; 1.46)** | 2.38 (2.22; 2.54) | 3.61 (3.22; 4.02) | 1.25 (1.05; 1.44)** |
| Uzbekistan | 5.60 (4.91; 6.37) | 8.53 (7.63; 9.36) | 1.41 (1.26; 1.54)** | 41.10 (33.32; 49.40) | 56.36 (64.83; 46.57) | 1.09 (0.91; 1.28)** | 1.28 (1.00; 1.73) | 1.57 (1.37; 1.79) | 1.06 (0.85; 1.27)** |
| Kyrgyzstan | 5.66 (5.04; 6.28) | 5.52 (4.89; 6.24) | -0.32 (-0.46; -0.18)** | 43.87 (36.69; 52.10) | 41.47 (34.88; 49.48) | -0.31 (-0.44; -0.18)** | 2.05 (1.79; 2.40) | 1.48 (1.27; 1.69) | -0.91 (-1.24; -0.58)** |
| Tajikistan | 5.68 (4.93; 6.41) | 7.35 (6.47; 8.22) | 0.83 (0.75; 0.91)** | 38.09 (30.42; 46.20) | 43.44 (35.39; 52.05) | 0.43 (0.31; 0.57)** | 3.34 (2.29; 5.50) | 2.21 (1.81; 2.67) | -1.29 (-1.58; -1.00)** |
| Turkmenistan | 3.74 (3.27; 4.28) | 5.88 (5.25; 6.51) | 1.53 (1.36; 1.72)** | 29.11 (24.10; 35.77) | 44.57 (37.55; 44.57) | 1.58 (1.41; 1.76)** | 1.22 (1.15; 1.29) | 1.96 (1.56; 2.38) | 1.53 (1.27; 1.79)** |

*** P-value <0.001*

Supplemental Table 2. Outputs of Frequentist Single-Level Regression Models.

| **Coefficient** | **Kazakhstan** | | **Uzbekistan** | | **Kyrgyzstan** | | **Tajikistan** | | **Turkmenistan** | |
| --- | --- | --- | --- | --- | --- | --- | --- | --- | --- | --- |
|  | **Slope (95%CI)** | **p-value** | **Slope (95%CI)** | **p-value** | **Slope (95%CI)** | **p-value** | **Slope (95%CI)** | **p-value** | **Slope (95%CI)** | **p-value** |
| Year | 1.40 (0.99, 1.81) | <0.001 | 1.49 (0.86, 2.14) | <0.001 | 1.13 (0.99, 1.29) | <0.001 | 1.78 (0.73, 2.84) | 0.01 | 1.87 (0.12, 3.63) | 0.03 |
| Population size | -0.15 (-2.48, 2.18) | 0.89 | -2.39 (-3.42, -1.36) | <0.001 | -18.66 (-21.25, -16.07) | <0.001 | -17.35 (-25.35, -9.35) | <0.001 | -18.85 (-36.93, -0.78) | 0.03 |
| Life expectancy | -0.71 (-1.52, 0.12) | 0.09 | 1.29 (0.53, 2.05) | 0.01 | -0.27 (-0.71, 0.16) | <0.001 | -0.33 (-0.83, 0.16) | 0.17 | -2.39 (-4.71, -0.07) | 0.04 |
| Employment in agriculture | -0.42 (-0.44, 1.27) | 0.33 | 0.60 (0.32, 0.88) | <0.001 | -0.16 (-0.22, -0.12) | 0.21 | -1.14 (-1.70, -0.59) | <0.001 | -1.14 (-1.62, -0.67) | <0.001 |
| Aged 65 and above | 3.83 (-5.77, -0.69) | 0.01 | -2.35 (-3.95, -0.76) | 0.01 | 4.81 (3.80, 5.80) | <0.001 | 9.95 (4.26, 15.66) | 0.01 | 2.97 (-0.03, 5.97) | 0.05 |
